# Early Diagnosis of Wilson’s Disease in Children in Southern China by Using Common Parameters

**DOI:** 10.3389/fgene.2022.788658

**Published:** 2022-02-10

**Authors:** Jianli Zhou, Qiao Zhang, Yuzhen Zhao, Moxian Chen, Shaoming Zhou, Yongwei Cheng

**Affiliations:** ^1^ Department of Gastroenterology, Shenzhen Children’s Hospital, Shenzhen, China; ^2^ Co-Innovation Center for Sustainable Forestry in Southern China, Key Laboratory of National Forestry and Grassland Administration on Subtropical Forest Biodiversity Conservation, College of Biology and the Environment, Nanjing Forestry University, Nanjing, China

**Keywords:** children, southern China, hepatolenticular degeneration, clinical features, genetic mutation

## Abstract

**Objective:** The aim of the study was to develop the early diagnostic criteria for Wilson’s disease (WD) in young children in southern China by using alanine aminotransferase (ALT) elevation as the first manifestation.

**Methods:** A cross-sectional retrospective analysis of the clinical data and genetic test results of children with WD in southern China in the past 4 years and the follow-up of their short-term prognosis were performed in this study.

**Results:** A total of 30 children (5.08 ± 2.06 years old) with elevated ALT as the first manifestation of WD in southern China were enrolled in this study, including 14 females and 16 males. Specifically, in all of the 30 cases (100%), the serum ceruloplasmin (CP) level was decreased, whereas the 24-h urinary copper level was increased. The genetic mutation test of the *ATP7B* gene was used to confirm the diagnosis. In particular, the two mutation sites, including p.R778L and p.I1148T, had the highest mutation frequencies, approximately 23.0 and 10.7%, respectively. Through follow-up, most of the children had good recovery.

**Conclusion:** Early diagnosis and treatment of WD would substantially increase the survival rate and have a better prognosis. In addition, in 5-year-old children from southern China, early diagnosis could be performed quickly by referring to the following three parameters: elevated ALT, decreased ceruloplasmin level, and increased 24-h urinary copper level. It lays a foundation for further studies with a larger sample size.

## 1 Introduction

Hepatolenticular degeneration (HLD), also known as Wilson’s disease (WD; OMIM 277900), is an autosomal recessive disorder of copper metabolism (Huster, 2010; Meranthi et al., 2020). The disease occurs all over the world, and the incidence rate in the human population is about 1:1,500–13,000 in East Asia and 1:7,000 in the United Kingdom (Chen et al., 2019; Xiao et al., 2019; Meranthi et al., 2020). The clinical features of WD include liver function injury, nervous system damage, psychiatric abnormality, corneal Kayser–Fleischer (K–F) ring, and decreased serum ceruloplasmin (Xiao et al., 2019). The onset age of WD ranges from infancy to more than 70 years, with an average age of 15.9 years (Xiao et al., 2019). Late diagnosis and treatment or irregular medication of WD could lead to irreversible brain damage or even death. Therefore, early diagnosis and treatment are crucial to reduce the irreversible sequelae of WD (Xiao et al., 2019).

WD can result from the mutation of the *ATP7B* (OMIM 606882) gene that encodes the intracellular copper transporter on chromosome 13, leading to an impaired intracellular copper output (Meranthi et al., 2020). *ATP7B* is a P-type ATPase and is mainly expressed in the liver. It binds copper to its N-terminal domain and is responsible for the transport of copper across the membrane, using ATP as its energy source. Studies have demonstrated that mutations at different sites can affect ATPase activity. Until now, more than 1,000 different mutations of *ATP7B* have been found in patients with WD in the Human Gene Mutation Database (HGMD v2021.11) (Stenson et al., 2017). The mutation of the *ATP7B* gene in WD affects the interaction between copper ions and ceruloplasmin and subsequent copper excretion in bile, which is the major way of excreting liver copper. If the copper excretion from bile is reduced, copper is then deposited in places around the liver, causing damage to hepatocytes. In addition, it results in elevated ALT as the primary clinical manifestation. Gradually, copper accumulates in the brain, cornea, and kidneys, causing damage to the corresponding organs and accompanying clinical symptoms (European Association for Study of Liver, 2012). Over time, the liver becomes progressively damaged by copper deposits, and some patients end up with cirrhosis or liver failure, as well as severe nervous and blood system damage (European Association for Study of Liver, 2012). Therefore, early diagnosis with high accuracy is crucial for patients with WD and their prognosis. To this end, the clinical features and genetic characteristics of 30 children diagnosed with WD and treated at Shenzhen Children’s Hospital in the past 4 years were analyzed in this study.

## 2 Material and Methods

### 2.1 Clinical Data Collection

This was a single-center cross-sectional retrospective study of 30 patients with WD with elevated ALT as their first manifestation in southern China, from May 2016 to May 2020. Medical history, physical examination, laboratory examination, and imaging findings were all collected as clinical data. Physical examination included jaundice, liver enlargement, K–F ring, and neurological symptoms. Laboratory tests included blood routine, hepatic, renal and immunological function tests, virology tests (hepatitis A, B, C, D, E, cytomegalovirus, and EB virus), ceruloplasmin, and 24-h urinary copper level. Imagological examinations included abdominal (liver) ultrasound, cardiac Doppler ultrasonography, and brain magnetic resonance imaging (MRI).

### 2.2 Genetic Data Collection

All of the cases were tested with *ATP7B* targeted gene panel sequencing (TGPS) or whole-exome sequencing (WES). The venous blood (2–5 ml) of the patient was drawn after the results of the serum ceruloplasmin (CP) level and 24-h urinary copper level were available, together with 5 ml of parental venous blood for comparison to verify the source of its pathogenic genes. All test protocols, including DNA extraction, construction of gene library, high-throughput sequencing, data analysis, Sanger sequencing verification, and bioinformatics analysis, were carried out by commercial companies such as BGI (The Beijing Genomics Institute, Shenzhen) and Mykino (Beijing).

### 2.3 Follow-Up Visit

All of the cases were carried out for follow-up studies using outpatient and telephone recordings, including examination, treatment, and outcome.

## 3 Results

### 3.1 Clinical Features (Table 1)

#### 3.3.1 Study Data

The subjects of the present study included 14 female and 16 male patients who were asymptomatic only with an elevated hepatase level. The minimum diagnosed age was 2 years, and the oldest patient was 11 years and 4 months old, with an average age of 5.08 ± 2.06 years. The average duration from the discovery of abnormal liver function to diagnosis was about 4 months, and the longest duration was 4 years and 5 months.

**TABLE 1:** Detailed clinical information of 30 children with WD.

Case	Gender	Onset age (years)	Neurological symptoms	Corneal K–F ring	Hb (g/L)	ALT (IU/L)	AST (IU/L)	AST/ALT	TB (umol/L)	CP (mg/dl)	24-h urinary copper (μg/24 h)	Mutations of *ATP7B*	Zygotic type
Case 1	Female	4.5	No	No	118	212	113	0.53	3.3	2.8	82.3	c.2975C > T (p.P992L)	Het
												c.2333G > T (p.R778L)	Het
Case 2	Female	5.33	No	No	127	161	116	0.72	6.7	5.3	80.7	c.2804C > T (p.T935M)	Het
												c.2333G > T (p.R778L)	Het
												c.2310C > G (p.L770L)	Het
Case 3	Female	4	No	No	121	358	222	0.62	13.4	4.5	85.2	c.2662A > C (p.T888P)	Het
												c.2333G > T (p.R778L)	Het
												c.2310C > G (p.L770L)	Het
Case 4	Male	5	No	No	131	92	85	0.92	3.6	7.5	111.2	c.314C > A (p.S105*)	Het
												c.2975C > T (p.P992L)	Het
Case 5	Male	6.58	No	No	129	135	81	0.6	14.7	3.5	121.2	c.2662A > C (p.T888P)	Het
												c.2268G > A (p.A756A)	Het
Case 6	Male	3.5	No	No	130	233	150	0.64	10.7	4.8	105.3	c.2804C > T (p.T935M)	Het
												c.3809A > G (p.N1270S)	Het
Case 7	Female	2.91	No	No	128	289	212	0.73	8	2.2	51.1	c.2662A > C (p.T888P)	Het
												c.2333G > T (p.R778L)	Het
Case 8	Male	3	No	No	128	151	106	0.7	5.4	5.4	123.9	c.2755C > G (p.R919G)	Het
												c.2333G > T (p.R778L)	Het
Case 9	Female	5.83	No	No	127	320	167	0.52	9.6	4.4	189.1	c.3316G > A (p.V1106I)	Het
												c.525dupA	Het
Case 10	Male	3.75	No	No	124	673	371	0.55	4.9	4.1	187.3	c.3426G > C (p.Q1142H)	Hom
												c.3443T > C (p.I1148T)	Hom
Case 11	Female	3.66	No	No	148	348	222	0.64	13.7	9	83.4	c.2662A > C (p.T888P)	Het
												c.3587A > G (p.D1196G)	Het
Case 12	Female	2.91	No	No	125	348	135	0.39	4.3	2	92.3	c.3244-2A > G	Het
												c.3426G > C (p.Q1142H)	Het
												c.3443T > C (p.I1148T)	Het
Case 13	Male	3.66	No	No	115	389	259	0.67	8.8	3.8	54.5	c.2975C > T (p.P992L)	Het
												c.2320_2321insTTGCCCAGGGCA	Het
Case 14	Male	3.33	No	No	139	440	243	0.55	7.5	4	191.4	c.3443T > C (p.I1148T)	Het
												c.4064G > A (p.G1355D)	Het
Case 15	Female	4.58	No	No	117	117	112	0.96	7.3	6.9	308.4	c.2975C > T (p.P992L)	Het
												c.3443T > C (p.I1148T)	Het
Case 16	Female	0.91	No	No	132	248	175	0.71	8.6	2.1	64.1	c.1470C > A (p.C490*)	Het
												c.3532A > G (p.T1178A)	Het
Case 17	Female	4.16	No	No	110	181	101	0.56	11.5	2.9	87.6	c.3220G > A (p.A1074T)	Het
												c.2333G > T (p.R778L)	Het
Case 18	Male	3	No	No	125	393	240	0.61	8.4	4.7	146.7	c.3220G > A (p.A1074T)	Het
												c.2333G > T (p.R778L)	Het
Case 19	Female	4.25	No	No	118	115	90	0.78	5.2	2.1	308.9	c.2145C > A (p.Y715*)	Het
												c.2333G > T (p.R778L)	Het
Case 20	Female	3	No	No	127	261	155	0.59	7.4	2.4	150.9	c.2333G > T (p.R778L)	Het
												c.525dupA	Het
												c.2310C > G (p.L770L)	Het
Case 21	Male	5.25	No	No	137	519	360	0.69	17.6	6	134.7	c.3532A > G (p.T1178A)	Het
												c.3443T > C (p.I1148T)	Het
Case 22	Male	3.25	No	No	130	596	669	1.12	13.6	6.1	145.7	c.3443T > C (p.I1148T)	Het
												c.2333G > T (p.R778L)	Het
Case 23	Male	1.91	No	No	121	73	75	1.03	5.7	3.8	68.3	c.4059G > A (p.W1353*)	Het
												c.2621C > T (p.A874V)	Het
Case 24	Male	7.41	No	No	131	330	170	0.52	6.3	1.9	256.1	c.2621C > T (p.A874V)	Het
												c.2333G > T (p.R778L)	Het
Case 25	Male	6	No	No	128	453	231	0.51	5.6	2.7	422	c.2272A > G (p.R758G)	Het
												c.2333G > T (p.R778L)	Het
Case 26	Male	3.75	No	No	125	219	135	0.62	13.3	6.3	94.8	c.2139C > G (p.Y713*)	Het
												c.2755C > G (p.R919G)	Het
Case 27	Male	11.33	No	No	144	143	121	0.85	5.5	3.9	298.7	c.3443T > C (p.I1148T)	Het
												c.3809A > G (p.N1270S)	Het
Case 28	Female	3	No	No	127	117	89	0.76	5.2	3.3	93.1	c.2333G > T (p.R778L)	Het
												c.3452G > A (p.R1151H)	Het
Case 29	Male	1.91	No	No	119	287	208	0.72	5.8	4.5	139.2	c.2975C > T (p.P992L)	Het
												c.2333G > T (p.R778L)	Het
Case 30	Female	7.33	No	No	98	106	350	3.3	43	5.6	840.7	c.2333G > T (p.R778L)	Het
												c.4003G > C (p.G1335R)	Het
												c.525dupA	Het

Note: Hb, hemoglobin; ALT, alanine aminotransferase; AST, aspartic transaminase; TB, total bilirubin; CP, ceruloplasmin; Het, heterozygous; Hom, homozygous.

#### 3.3.2 Blood Biochemical Test

The blood test suggested that ALT was elevated in all of the patients, ranging from 73 to 673 IU/L. In particular, ALT of 11 cases (36.6%) was found to be slightly elevated (increased <5 (upper limit of normal, ULN) times the reference value). In addition, the ALT levels of 14 cases (46.6%) were moderately elevated, that is, 5–10 ULN, whereas 5 cases (16.6%) were found to have severely elevated ALT levels (>10 ULN). Furthermore, the aspartic transaminase (AST) of patients ranged from 75 to 669 IU/L. Specifically, 27 cases (90%) were found to have an AST/ALT ratio of less than 1. In addition, only three cases (10.0%) showed an AST value higher than that of ALT, and the one with an AST/ALT of >2 had jaundice, coagulation dysfunction, liver failure, and eventually died.

#### 3.3.3 Corneal Kayser–Fleischer (K–F) Ring

The 30 children were examined by an ophthalmologist. No corneal K–F ring was found, indicating that there was no eye damage in this group.

#### 3.3.4 Performance of the Nervous System

All of the 30 children had no neurological symptoms. In this group, 12 children underwent brain MRI, and none of them found abnormalities in the basal ganglia, thalamus, and brainstem.

#### 3.3.5 Indicators of Copper Metabolism

The CP level was reduced in all of the 30 cases (100.00%), and the detection value was less than 10 mg/dl. Furthermore, the 24-h urinary copper level was increased in all 30 cases and was more than 40 μg/24 h. In particular, 4 cases (13.3%) reached 40–80 μg/24 h, and the remaining 26 cases (86.6%) were more than 80 μg/24 h.

### 3.2 Genetic Analysis (Table 2)

**FIGURE 1:**
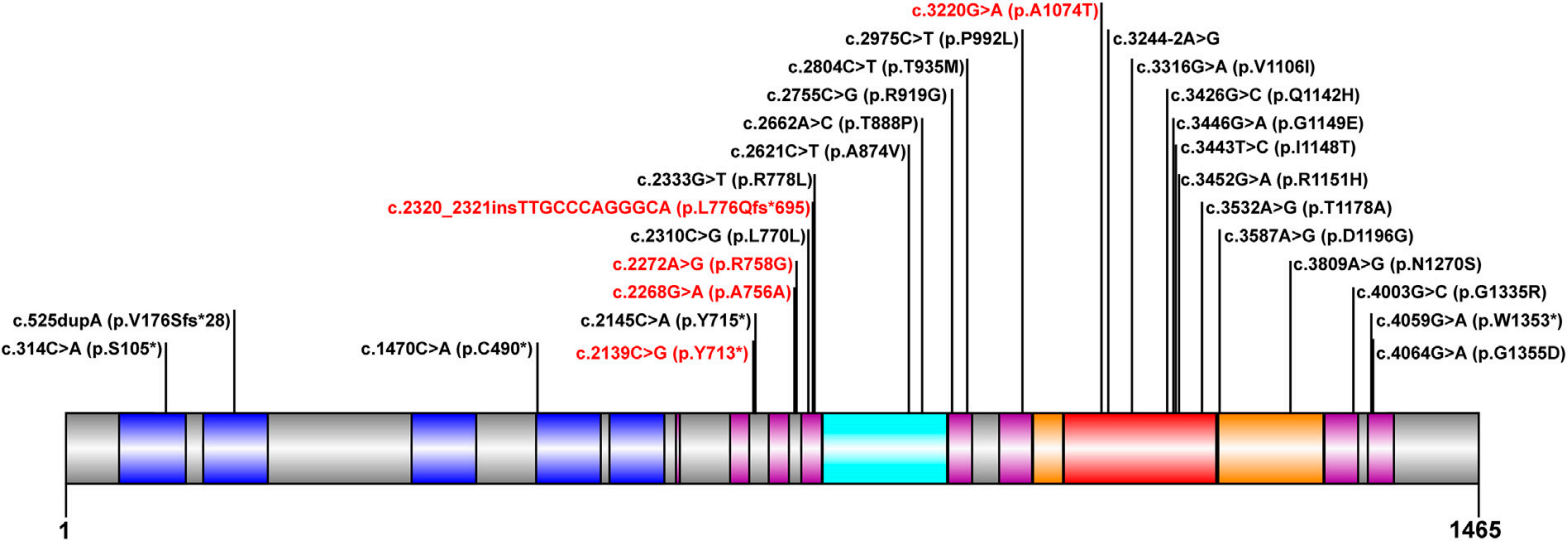
Scheme of ATP7B with functional regions and mutations reported in our cohort. The metal binding unit, transmembrane domain, actuator domain, phosphorylation domain, and nucleotide-binding domain are colored in blue, purple, cyan, orange, and red, respectively. The novel mutations detected in this study are in red.

The mutation analysis of the *ATP7B* gene was performed for all of the 30 children, and a total of 65 allelic mutations were detected. This included 51 missense mutations (78.4%), 5 nonsense mutations (7.6%), 4 synonymous mutations (6.1%), 4 frameshift mutations (6.1%), and 1 splicing mutation (1.5%). In our study, there were a total of 28 mutation sites, including 23 reported mutation sites and 5 novel mutation sites. These 28 loci were distributed among different functional regions, including the metal binding units (MBUs), transmembrane domain (TM), actuator domain (A-domain), phosphorylation domain (P-domain), and nucleotide-binding domain (N-domain) (Figure 1). The mutation hot spot was identified as p.R778L, including 15 (23.0%) mutation sites at this spot. Furthermore, the second popular mutation site was p.I1148T, which occurred in 7 (10.7%) patients. The five novel mutations included c.2139C > G (p.Y713*), c.2268G > A (p.A756A), c.2272A > G (p.R758G), c.2320_2321insTTGCCCAGGGCA (p.L776Qfs*695), and c.3220G > A (p.A1074T). Variants p. Y713* and p. L776Qfs*695 can be interpreted as “likely pathogenic” according to the American College of Medical Genetics and Genomics (ACMG) standard (PVS1_strong + PM2+PP3), while the other three mutations can be classified as “variants with uncertain clinical significance” (PM2+PP3) (Richards et al., 2015).

**TABLE 2:** Information of *ATP7B* gene mutations in 30 children with WD.

Mutations in *ATP7B* gene	Location	Functional region	Mutation type	Number of mutations	Frequency of mutations (%)	Novelty
c.2333G > T (p.R778L)	Exon 8	TM4	Missense	15	23.00	
c.3443T > C (p.I1148T)	Exon 16	ATP loop	Missense	7	10.70	
c.2975C > T (p.P992L)	Exon 13	TM6	Missense	5	7.60	
c.2662A > C (p.T888P)	Exon 11	ATPase	Missense	4	6.10	
c.525dupA (p.V176Sfs*28)	Exon 2	Cu2	Fame-shift	3	4.60	
c.2310C > G (p.L770L)	Exon 8	TM4	Synonymous	3	4.60	
c.2621C > T (p.A874V)	Exon 11	ATPase	Missense	2	3.00	
c.2755C > G (p.R919G)	Exon 12	TM5	Missense	2	3.00	
c.2804C > T (p.T935M)	Exon 12	TM5	Missense	2	3.00	
c.3426G > C (p.Q1142H)	Exon 16	ATP loop	Missense	2	3.00	
c.3532A > G (p.T1178A)	Exon 16	ATP loop	Missense	2	3.00	
c.3809A > G (p.N1270S)	Exon 18	ATP hinge	Missense	2	3.00	
c.314C > A (p.S105*)	Exon 2	Cu1	Nonsense	1	1.50	
c.1470C > A (p.C490*)	Exon 3	Cu5	Nonsense	1	1.50	
c.2139C > G (p.Y713*)	Exon 8	TM2/TM3	Nonsense	1	1.50	Novel
c.2145C > A (p.Y715*)	Exon 8	TM2/TM3	Nonsense	1	1.50	
c.2268G > A (p.A756A)	Exon 8	TM3/TM4	Synonymous	1	1.50	Novel
c.2272A > G (p.R758G)	Exon 8	TM3/TM4	Missense	1	1.50	Novel
c.2320_2321insTTGCCCAGGGCA (p.L776Qfs*695)	Exon 8	TM4	Fame-shift	1	1.50	Novel
c.3220G > A (p.A1074T)	Exon 14	ATP loop	Missense	1	1.50	Novel
c.3244-2A > G	Exon 15	ATP loop	Splicing	1	1.50	
c.3316G > A (p.V1106I)	Exon 15	ATP loop	Missense	1	1.50	
c.3446G > A (p.G1149E)	Exon 16	ATP loop	Missense	1	1.50	
c.3452G > A (p.R1151H)	Exon 16	ATP loop	Missense	1	1.50	
c.3587A > G (p.D1196G)	Exon 17	ATP hinge	Missense	1	1.50	
c.4003G > C (p.G1335R)	Exon 19	TM7	Missense	1	1.50	
c.4059G > A (p.W1353*)	Exon 20	TM8	Nonsense	1	1.50	
c.4064G > A (p.G1355D)	Exon 20	TM8	Missense	1	1.50	

Note: WD, Wilson’s disease; Cu, metal-binding domain; TM, transmembrane domain; ATPase, copper (or silver)-translocating P-type ATPase, domain.

### 3.3 Criteria of Disease Diagnosis

The diagnosis criteria for WD were according to EASL Clinical Practice Guidelines: Wilson’s disease, from the European Association for Liver Research in 2012 (European Association for Study of Liver, 2012). The parameters used in this evaluation are listed as follows:

(1) Kayser–Fleischer ring (2 points);
(2) neuropsychiatric symptoms suggestive of WD (severe: 2 points and moderate: 1 point);
(3) serum ceruloplasmin content (normal value or >20 mg/dl) normal (0 point), 10–20 mg/dl (1 point), and <10 mg/dl (2 points);
(4) Coombs negative hemolytic anemia with elevated serum copper (1 point);
(5) quantitative determination of liver copper: normal (−1 point), not more than 5 ULN (1 point), and greater than 5 ULN (2 points). Rhodanine staining of hepatocytes is positive (if the quantitative determination of liver copper cannot be obtained) (1 point);
(6) urine copper in the absence of acute hepatitis: normal (0 points), which is 1–2 ULN (1 point), more than 2 ULN (2 points), application of 2 doses of 0.5 g D-penicillamine, and the copper content is more than 5 ULN (2 points);
(7) analysis of gene mutation: pathogenic mutations on both chromosomes (4 points), pathogenic mutations on a single chromosome (1 point), and no pathogenic mutations (0 points).

If the total score is ≥4 points, the possibility of WD is high; if the score is 3 points, it is likely to be WD, but more tests are needed (liver biopsy is required); and if the score is ≤2 points, it is unlikely to be WD. According to the aforementioned criteria, all of the 30 cases were ≥4 points (without checking for liver copper level, the score was from 7 to 9) (Table 3). Since the liver biopsy was invasive, and their parents did not agree to do it, no liver biopsy was performed. Even if the liver biopsy is normal, we subtract one point, and it is still more than four points. Therefore, all of the 30 cases could be diagnosed as WD, and the genetic results confirmed our diagnosis.

**TABLE 3:** Diagnosis score of the cases with WD.

Case	Corneal K–F ring	Neurologic symptoms	CP	Coombs-negative hemolytic anemia	Liver copper	24-h urinary copper	Mutation analysis	Total score
Case 1	0	0	2	0	−	2	4	8
Case 2	0	0	2	0	−	2	4	8
Case 3	0	0	2	0	−	2	4	8
Case 4	0	0	2	0	−	2	4	8
Case 5	0	0	2	0	−	2	4	8
Case 6	0	0	2	0	−	2	4	8
Case 7	0	0	2	0	−	1	4	7
Case 8	0	0	2	0	−	2	4	8
Case 9	0	0	2	0	−	2	4	8
Case 10	0	0	2	0	−	2	4	8
Case 11	0	0	2	0	−	2	4	8
Case 12	0	0	2	0	−	2	4	8
Case 13	0	0	2	0	−	1	4	7
Case 14	0	0	2	0	−	2	4	8
Case 15	0	0	2	0	−	2	4	8
Case 16	0	0	2	0	−	1	4	7
Case 17	0	0	2	0	−	2	4	8
Case 18	0	0	2	0	−	2	4	8
Case 19	0	0	2	0	−	2	4	8
Case 20	0	0	2	0	−	2	4	8
Case 21	0	0	2	0	−	2	4	8
Case 22	0	0	2	0	−	2	4	8
Case 23	0	0	2	0	−	1	4	7
Case 24	0	0	2	0	−	2	4	8
Case 25	0	0	2	0	−	2	4	8
Case 26	0	0	2	0	−	2	4	8
Case 27	0	0	2	0	−	2	4	8
Case 28	0	0	2	0	−	2	4	8
Case 29	0	0	2	0	−	2	4	8
Case 30	0	0	2	1	−	2	4	9

Note: WD, Wilson’s disease; K–F ring, Kayser–Fleischer ring; CP, ceruloplasmin; -, no data.

### 3.4 Follow-Up Record and Prognosis Evaluation

**FIGURE 2:**
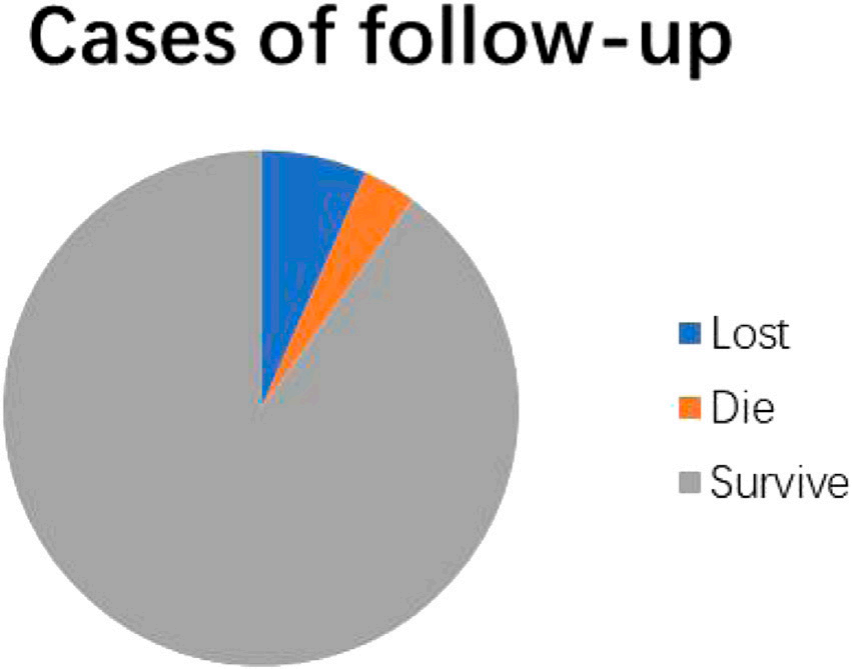
Pie chart of statistics in follow-up record and prognosis evaluation.

The follow-up time was from 1 month to 4 years and 2 months after diagnosis. In detail, 28 of 30 cases (93.3%) were successfully recorded during the follow-up, and the other 2 cases were lost. In particular, 27 cases used basic treatment, including a low-copper diet, oral zinc preparations, and vitamins B, while 25 of them were treated with penicillamine. In total, 27 cases (90%) survived and had good recovery of liver function during the course of treatment, while 20 cases (20/25) were still treated with penicillamine and treatment for 5 cases was stopped. However, 1 child (3.3%) died of acute liver failure (Figure 2).

## 4 Discussion

The clinical manifestations of children with WD may be diverse due to the starting time of treatment (Lorincz, 2010; Moores et al., 2012). In general, the copper excretion mechanism is not yet fully developed in newborn babies and becomes more effective within the first year after their birth. However, the key pathways of copper excretion in patients with WD fail to develop or have dysfunction, which leads to copper accumulation during the patient’s life, gradually producing various clinical symptoms (Manolaki et al., 2009). Although WD is diagnosed in patients aged 5–35 years (mean, 13 years) (Lin et al., 2014), younger and older patients (>70) are also diagnosed (Stremmel et al., 1991; European Association for Study of Liver, 2012; Lin et al., 2014; Wiernicka et al., 2017). A study of 143 children with WD showed that 21 (15%) of them developed abnormal liver function before the age of 5 years (Wiernicka et al., 2017). At an average age of 9–13 years, the most common initial presentation of children with WD is liver disease (Saito, 1987; Walshe, 1989). Moreover, about 8–10% of children with WD have chronic active hepatitis (Gitlin, 2003). As our cases were from 2 years old to 11 years and 4 months old (5.08 ± 2.06 years), all of them were asymptomatic and just showed up with elevated ALT. Previously, Japanese researchers have suggested that ALT could be the first parameter to screen children with WD between the ages 4 and 8 years (Hayashi et al., 2019), which was similar to our cases. In a previous study involving children and adults, liver presentation was more common in female patients, while neurological presentation was more common in male patients (Ferenci et al., 2019). However, our cases had hepatic presentations only, and the male/female relationship with WD needs to be further investigated in children.

Furthermore, in our group, no corneal K–F ring was detected, making it significantly different from the cases aged 20–30 years old reported in other studies (Bandmann et al., 2015). In addition, older patients (>15 years) are more likely to be diagnosed with neurological manifestations (Oder et al., 1991). The most common age at which WD develops neurological symptoms is 15–21 years (Saito, 1987; Oder et al., 1991; Lorincz, 2010; Žigrai et al., 2020). The discrepancy in the age of WD onset probably reflects variations in gene mutation and penetrance, extragenic factors, and other environmental factors (e.g., diet) (Ala and Schilsky, 2004). However, our cases were younger and were not accompanied by neurological manifestations, and 12 cases of the brain MRI were all negative. We believe that these young children with WD without neurological symptoms do not need to be routinely evaluated by brain MRI.

According to previous reports, the biochemical examination showed that 69.8% of patients with WD have low serum CP, and a serum CP of less than 20 mg/dL has very good accuracy in diagnosing WD (Kim et al., 2015). Furthermore, low CP had a sensitivity of 77–99% and a specificity of 55–88.2% (Ryan et al., 2019). In addition, research reported that 24-h urinary copper levels were increased in all patients (100%), and a level higher than 100 μg/24 h was useful for diagnosing WD (Vieira et al., 2012). In our study, all of the cases had high urinary copper levels (more than 40 μg/24 h) and low serum CP (less than 10 mg/dl). Meanwhile, a high urinary copper level and low serum CP had good diagnostic accuracy for WD (Aksu et al., 2018). Therefore, our results also support this argument.

WD is caused by homozygous or compound heterozygous mutations within *ATP7B*. At present, the human gene mutation database has more than 1,000 mutations of the *ATP7B* gene reported, including missense/nonsense mutations, splice site mutations, small deletion/insertion mutations, and frameshift mutations. Mutations can occur anywhere in the gene, including exons, introns, and even promoter regions (Coffey et al., 2013). Furthermore, the mutations of the *ATP7B* gene have genetic heterogeneity in different races and regions. For example, the most common type of mutation in the European population is p.H1069Q, which is more common in Italy, Sweden, and Romania, with an allele frequency ranging from 30 to 70% (Folhoffer et al., 2007). Contrastingly, the most common type of mutation in the Asian population is the missense mutation p.R778L, which is also the most common mutation in China, South Korea, and Japan, with an allele frequency ranging from 17.3 to 60% (Okada et al., 2000; Liu et al., 2004). Besides p.R778L, other high-frequency mutations include p.P992L and p.Q1399R. Similarly, our study also found that the most abundant mutation type was the missense mutation p.R778L, accounting for 23.0% of the total cases. In addition, the second abundant mutation type in our study was the missense mutation p.I1148T (10.7%). Whether this mutation type is representative of children in southern China remains to be investigated. However, the third abundant mutation type was the missense mutation p.P992L (7.6%), showing consistency with known Asian mutation frequencies. In our study, different gene mutations (affecting different functional domains) of the cases had almost identical clinical phenotypes, which were similar to the previous study (Ferenci et al., 2019). Only one patient died, whose *ATP7B* had three heterozygous mutations, including c.2333G > T (p.R778L), c.4003G > C (p.G1335R), and c.525dupA, and they included two missense mutations and one frameshift mutation, which affected the functional regions of MBU2, TM4, and TM7. Therefore, the more functional domains are affected, the worse the prognosis may be.

At present, the diagnosis of WD mainly relies on typical clinical manifestations, laboratory tests, and genetic testing (Huster, 2010). Early diagnosis and intervention are essential to delay the progression of the disease and prevent irreversible sequelae. In our study, 30 cases were diagnosed with an elevated ALT level as the first symptom, together with a decreased CP level, an increased 24-h urinary copper level, and *ATP7B* mutations, suggesting that these three parameters (namely, elevated ALT, decreased CP level, and increased 24-h urinary copper level) are closely related to the early diagnosis of WD in about 5-year-old children in southern China. Thus, we propose that the combined detection of elevated ALT, decreased ceruloplasmin level, and increased 24-h urinary copper level can be useful for an early diagnosis of WD in about 5-year-old asymptomatic children in southern China. In recent years, some researchers thought that genetic screening following serum CP testing reduced costs and facilitated prioritization of non-invasive methods for definitive diagnosis, as well as in asymptomatic or family history cases (Barada et al., 2017; García-Villarreal et al., 2021). Furthermore, other researchers believed that the serum CP level, 24-h urinary copper excretion, and K–F rings could be used to identify patients with WD (Dong et al., 2021). Patients with serum CP levels below 12 mg/dl and children with urinary copper excretion above 40 µg/24 h should undergo genetic testing for WD. As WD needs to identify the diseases, namely, Menkes disease, occipital horn syndrome (OHS), Indian childhood cirrhosis (ICC), and some other diseases and in specific subgroups defined by age, ethnicity, or clinical subgroups, our three parameters (elevated ALT, decreased CP level, and increased 24-h urinary copper level) may not be suitable (Lorincz, 2018; Ryan et al., 2019). However, they can be useful for the early diagnosis of WD in about 5-year-old asymptomatic children in southern China.

Until now, WD was one of the few genetic diseases that could be controlled. The treatment principles are early diagnosis and treatment, lifetime care, and personalized protocol. Current treatment measures include drug therapy, surgical treatment, gene and cell therapy, and rehabilitation (Wiggelinkhuizen et al., 2009). Currently, penicillamine is one of the classic drugs for the treatment of hepatolenticular degeneration due to its effectiveness and cheap price. Studies have shown that certain molecular chaperone drugs (such as 4-phenylbutyric acid) and p38 and JNK inhibitors can correct the mislocalization of the mutant protein and restore the transport function of this protein (Mulligan and Bronstein, 2020). Furthermore, the small-molecule DPM-1001 can effectively reduce the copper deposition in the liver and the brain in the hepatolenticular degeneration mouse model (Krishnan et al., 2018). In particular, personalized cell and (or) gene therapy is the current research hot spot. Its fundamental purpose is to restore the function of *ATP7B*-mediated hepatic and bile duct excretion of copper (Murillo et al., 2016), and it may be the most promising treatment in the future. The incidence of acute liver failure in WD has previously been reported to be 15–47% (Das et al., 2021; Devarbhavi et al., 2014; Rukunuzzaman, 2015). Among the successful follow-up cases in this group, except for one case (3.3%) with liver failure, the liver function recovered well after the application of penicillamine, oral zinc preparations, B vitamins, and low-copper diet, etc. It showed that as long as early diagnosis and early treatment had been applied, there would be good clinical results in prognosis for children carrying genetic mutations of the *ATP7B* gene. Our low incidence of acute liver failure may be related to sample size and duration of follow-up.

## 5 Conclusion

WD is an autosomal recessive genetic disease with diverse clinical manifestations. The group of patients reported in this study came from cities in southern China. Early diagnosis and treatment of WD would substantially increase the survival rate and have a better prognosis. All of these cases had elevated ALT, decreased ceruloplasmin content, and an elevated 24-h urinary copper level, indicating solid first manifestation and potential large-scale screening methods to diagnose WD at an early stage in 5-year-old asymptomatic children in southern China. Although this initial diagnosis can be further confirmed by using genetic testing of the *ATP7B* gene, it should be confirmed by further research with larger sample sizes.

## Data Availability

The datasets for this article are not publicly available due to concerns regarding participant/patient anonymity. Requests to access the datasets should be directed to the corresponding authors.

## Abbreviations

ALT, alanine aminotransferase; ALP, alkaline phosphatase; AST, aspartic transaminase; BGI, Beijing Genomics Institute; CP, ceruloplasmin; EEG, electroencephalogram; EASL, European Association for the Study of the Liver; FIB, fibrinogen; GGT, gamma-glutamyl transpeptidase; HLD, hepatolenticular degeneration; INR, international normalized ratio; K–F, Kayser–Fleischer; MRI, magnetic resonance imaging; PT, prothrombin time; ULN, upper limit of normal; WES, whole-exome sequencing; WD, Wilson’s disease.
